# Biochemical Characterization of Two Rhamnogalacturonan Lyases From *Bacteroides ovatus* ATCC 8483 With Preference for RG-I Substrates

**DOI:** 10.3389/fmicb.2021.799875

**Published:** 2022-01-11

**Authors:** Weiyang Wang, Yibing Wang, Haoting Yi, Yang Liu, Guojing Zhang, Le Zhang, Kevin H. Mayo, Ye Yuan, Yifa Zhou

**Affiliations:** ^1^Engineering Research Center of Glycoconjugates, Ministry of Education, Jilin Provincial Key Laboratory of Chemistry and Biology of Changbai Mountain Natural Drugs, School of Life Sciences, Northeast Normal University, Changchun, China; ^2^Department of Biochemistry, Molecular Biology and Biophysics, University of Minnesota, Minneapolis, MN, United States

**Keywords:** rhamnogalacturonan lyase, polysaccharide lyase family 11, *Bacteroides ovatus*, type I rhamnogalacturonan, rhamnogalacturonan oligosaccharides

## Abstract

Rhamnogalacturonan lyase (RGL) cleaves backbone α-1,4 glycosidic bonds between L-rhamnose and D-galacturonic acid residues in type I rhamnogalacturonan (RG-I) by β-elimination to generate RG oligosaccharides with various degrees of polymerization. Here, we cloned, expressed, purified and biochemically characterized two RGLs (Bo3128 and Bo4416) in the PL11 family from *Bacteroides ovatus* ATCC 8483. Bo3128 and Bo4416 displayed maximal activity at pH 9.5 and pH 6.5, respectively. Whereas the activity of Bo3128 could be increased 1.5 fold in the presence of 5 mM Ca^2+^, Bo4416 required divalent metal ions to show any enzymatic activity. Both of RGLs showed a substrate preference for RG-I compared to other pectin domains. Bo4416 and Bo3128 primarily yielded unsaturated RG oligosaccharides, with Bo3128 also producing them with short side chains, with yields of 32.4 and 62.4%, respectively. Characterization of both RGLs contribute to the preparation of rhamnogalacturonan oligosaccharides, as well as for the analysis of the fine structure of RG-I pectins.

## Introduction

Pectin degrading enzymes are found in a variety of microorganisms (de Vries and Visser, 2001; Masoud and Jespersen, 2006; Martens et al., 2011; Tayi et al., 2016), and are widely used in the food industry to degrade pectin. There are three main classes of pectins: homogalacturonan (HG), type I rhamnogalacturonan (RG-I), and type II rhamnogalacturonan (RG-II) (Willats et al., 2001; Lerouxel et al., 2006; Atmodjo et al., 2013). Both HG and RG-II domains form their backbones *via* α-1,4-GalA linkages (Ridley et al., 2001; Mohnen, 2008), whereas the RG-I backbone is composed of repeating disaccharides of α-1,2-Rha and α-1,4-GalA, with neutral sugar side chains decorating the polymer at Rha O4 positions. These neutral sugar side chains usually consist of arabinans, galactans, arabinogalactans-I and arabinogalactans-II (Oechslin et al., 2003; Yapo et al., 2007).

Nowadays, many HG degrading enzymes derived from microorganisms have been extensively studied and used to clarify fruit juices and in other processes (Kashyap et al., 2001; Kc et al., 2020; Wu et al., 2020). In contrast, the number of reports on enzymes that cleave the RG-I backbone is considerably less extensive. Enzymes that degrade the RG-I backbone include rhamnogalacturonan hydrolases (RGHs) and rhamnogalacturonan lyases (RGLs) (Silva et al., 2016). Rhamnogalacturonan lyases (RGLs) are the enzymes that cleave the RG-I backbone by hydrolyzing α-1,4 glycosidic bonds between Rha and GalA by β-elimination to form oligosaccharides with α-4,5-unsaturated-GalA at non-reducing end (Azadi et al., 1995; Ochoa-Jiménez et al., 2018a). RGLs play an important role in carbon source acquisition of microorganisms. The RGLs in *Bacteroides thetaiotaomicron* VPI-5482 (BT4170, BT4175, and BT4183) can degrade RG-I with arabinosidases and galactosidases, so that *Bacteroides thetaiotaomicron* can use pectin as carbon source (Luis et al., 2018). RGL genes regulate growth and cohesion between different pectin polymers during plant development and softening during fruit ripening. For instance, the RGL gene participates in pollen tube germination, fruit firmness, and the fruit senescence phenomena that impact postharvest shelf life of tomato (Ochoa-Jiménez et al., 2018b). At present, RGLs have been classified into four polysaccharide-lyase (PL) families (PL4, PL9, PL11, and PL26) in the CAZy carbohydrate-active enzyme database^1^. RGLs can be divided into endo-RGL (EC 4.2.2.23) and exo-RGL (EC 4.2.2.24) according to random attacks or an attack on the terminal glycosidic bond, respectively. The first reported endo-RGL was RghB derived from *A. aculatus* and belonging to the PL4 family, followed by assessment of the mechanism of action and main products produced (Mutter et al., 1996, 1998; Jensen et al., 2010). Subsequently, other recombinant endo-RGLs were characterized, e.g., RhiE (Laatu and Condemine, 2003), YesW (Ochiai et al., 2007a), Rgl11A (McKie et al., 2001), Rgl11Y (Pagès et al., 2003), and CtRGLf (Dhillon et al., 2016) from bacteria; PcRGL4A (Iwai et al., 2015), AsRglA (Yoshino-Yasuda et al., 2012), AN7135.2, and AN6395.2 (Bauer et al., 2006) from fungi; *FchRGL1* (Méndez-Yañez et al., 2020) from plants. Among these reported enzymes, detailed structural analyses of their products are rarely mentioned.

*Bacteroides ovatus* is a good microorganism to explore new RG-I degrading enzymes. *Bacteroides ovatus* can be grown on structurally complex pectic substrates as their carbon source, e.g., spinach pectin (Centanni et al., 2020) or rhamnogalacturonan-enriched polysaccharide fractions. Rhamnogalacturon oligosaccharides are primarily degraded into RG disaccharides following incubation with *Bacteroides ovatus* (Van Laere et al., 2000). In the present study, we cloned, expressed, and characterized two PL11 family endo-RGL genes from *Bacteroides ovatus* ATCC 8483, and then evaluated their ability to degrade RG-I.

## Materials and Methods

### Strains, Plasmids, and Media

*Bacteroides ovatus* ATCC 8483 (China General Microbiological Culture Collection Center, Beijing, China) was cultured in an anaerobic chamber having an atmosphere of 95% N_2_ and 5% H_2_ with Trypticase Soy Broth medium. *Escherichia coli* BL21 (DE3) [Sangon Biotech (Shanghai) Co., Ltd., Shanghai, China] and the pET-28a (+) vector (Novagen, Madison, United States) were employed as host cells and expression vector, respectively. *Escherichia coli* BL-21 (DE3) transformants were grown in Luria-Bertani culture with 50 μg/ml kanamycin [Tiangen Biotech (Beijing) Co., Ltd., Beijing, China].

### Cloning and Expression of Bo3128 and Bo4416

To obtain mature proteins, the signal sequences of Bo3128 and Bo4416 were predicted using SignalP 5.0^2^. The nucleotide sequences encoding for Bo3128 and Bo4416 genes were amplified from the DNA of *Bacteroides ovatus* ATCC 8483, with the following primer sequences. Bo3128: 5′-GACTGGTGGACAGCAAATGGGTCGCGGATCCCAGGGAA AGCAACGCCAGAT-3′ and 5′- GATCTCAGTGGTGGTGGTG GTGGTGCTCGAGTCATTTCCCG-AACCGGCCAG-3,′ and Bo4416: 5′- GACTGGTGGACAGCAAATGGGT-CGCGGA TCCTGCTCGGATAATGACCCCGT-3′ and 5′- GATCTCAG TGGTGG-TGGTGGTGGTGCTCGAGTTATTCTGCTTTTTT CAAAG. The gene fragments and pET-28a (+) were then joined by seamless junctions using the Ready-to-Use Seamless Cloning Kit [Sangon Biotech (Shanghai) Co., Ltd., Shanghai, China], respectively. The recombinant plasmids were finally transfected into *Escherichia coli* BL21 (DE3) for expression. Cells harboring the recombinant plasmids were inoculated in 500 ml of Luria-Bertani medium containing 50 μg/ml kanamycin at 37°C and stirred at 180 rpm. The culture, induced with 0.5 mM IPTG, was grown for 24 h at 16°C until the OD_600 nm_ of the medium reached 0.6. To enhance production efficiency of the recombinant proteins, a 5 l Biotech-5BG bioreactor (Baoxing Bioengineering, China) was used for high-density fermentation to produce recombinant Bo3128 and Bo4416. The working fermentation volume was 3 l, and the inoculum was 5%. When the OD_600__*nm*_ of the medium reached 20, 0.5 mM IPTG was added to induce protein expression for 20 h.

### Preparation and Purification of Bo3128 and Bo4416

To harvest cells, cultures were centrifuged at 5,000 rpm for 15 min at 4°C, and the cells were resuspended in 20 ml lysis buffer (40 mM Tris–Hcl, pH 8.0, 0.1 M NaCl) and disrupted by sonication on ice for 15 min. Cell debris was removed by centrifugation at 13,000 rpm, 4°C for 30 min. The supernatant of cell lysate was then loaded onto a Ni-NTA column to obtain purified protein. The Ni-NTA column was equilibrated by 100 ml of lysis buffer, then the supernatant of cell lysate was applied onto Ni-NTA column. The impure proteins were washed by 100 ml washing buffer (40 mM Tris–Hcl, pH 8.0, 0.1 M NaCl, 20 mM imidazole), and the objective protein was collected with 50 ml elution buffer (40 mM Tris–Hcl, pH 8.0, 0.1 M NaCl, 300 mM imidazole). Finally, the purified protein was dialyzed to remove imidazole in buffer containing 40 mM Tris–Hcl (pH 8.0). Sodium dodecyl sulfate-polyacrylamide gel electrophoresis (SDS-PAGE) on 12% separating gel was used to analyze the purified enzymes Bo3128 and Bo4416.

### Enzyme Assay

The activities of Bo3128 and Bo4416 were determined with RG-I-AT. RG-I-AT is the RG-I type pectin extracted from the root of *Adenophora tetraphylla* (Thunb.) Fisch as described previously (Sun et al., 2019). Using a Tecan Spark 10 M microplate reader (Tecan Trading AG, Switzerland) by measuring the increase in optical density at 235 nm. The 200 μl reaction mixture containing 40 mM Tris–Hcl (pH 8.0), 1 mg/ml RG-I-AT, 5 mM CaCl_2_ and 10 μg purified protein was incubated at 37°C for 5 min. One unit of RGL activity was defined as the amount of enzyme required to generate 1 μmol of 4,5-unsaturated galacturonic acid in 1 min. The value of the molar extinction coefficient of the 4,5-unsaturated galacturonic acid at 235 nm was 5.5 × 10^3^ M^–1^ cm^–1^ (Kester and Visser, 1994). All assays were performed in triplicate.

### Biochemical Characterization

pH values for optimal Bo3128 and Bo4416 activities were measured using 1 mg/ml RG-I-AT substrate in 20 mM buffer [acetate buffer (pH 3.0–7.0), phosphate buffer (pH 7.0–9.0) and glycine-NaOH buffer (pH 9.0-11.0)] at 37°C. pH stabilities of the enzymes were determined by measuring the residual activity of the enzyme after incubating the purified protein at various pH values at 4°C for 24 h. The optimal temperature of the enzymes was determined in 20 mM phosphate buffer (pH 7.0) at different temperatures ranging from 20°C to 60°C. The temperature stability of Bo3128 and Bo4416 were examined by incubating the purified enzyme in 20 mM sodium phosphate buffer (pH 7.0) at different temperatures for 4 h. The effects of metal ions on the activity of Bo3128 and Bo4416 were determinated by detecting the absorbance of 235 nm after the proteins were incubated at 37°C in the presence of 5 mM and 50 mM metal ion. Experiments were performed in triplicate.

### Substrate Specificity

Rhamnogalacturonan I from potato pectic fiber (RG-I-P), galactan from Potato, and arabinan from sugar beet were purchased from Megazyme International Ireland Ltd. (Wicklow, Ireland). HG (48% Methyl-esterified, from Citrus Peel) was purchased from Sigma-Aldrich (St. Louis, MO, United States). De-HG was obtained by hydrolysis HG (48% Methyl-esterified) (Yu et al., 2021). RG-II was obtained from *Apocynum venetum* (Wu et al., 2018). The substrates were incubated with 1 mg/ml and 10 μg enzyme containing 5 mM CaCl_2_ in 200 μl 20 mM Tris–Hcl (pH 8.0) buffer at 37°C for 5 min. Substrate specificity was measured by detecting the change in absorbance at 235 nm. The experiments were conducted in triplicate.

### Kinetic Parameters

The 200 μl reaction solution contained different concentrations of RG-I-AT (0.01, 0.02, 0.04, 0.06, 0.08, 0.1, 0.2, 0.3, 0.4, 0.5, 0.6, 0.8, and 1 mg/ml) and 10 μg enzyme in a solution of 5 mM CaCl_2_, 20 mM Tris–Hcl buffer (pH 8.0) incubated at 37°C for 5 min. The absorbance of the reaction mixture was detected at 235 nm, and data were processed using Graph Pad 8.0 software and double-reciprocal (Lineweaver–Burk) plots. The kinetic parameters K_m_ and V_max_ were calculated by fitting the data with Michaelis-Menten kinetics. The k_cat_ was defined as the number of substrate molecules converted per enzyme molecule per second. The experiments were performed in triplicate.

### Seperation and Purification of Enzymatic Products

RG-I-AT was degraded by Bo3128 or Bo4416 in a ratio of 1 mg: 100 mU in 20 mM pH 8.0 Tris–Hcl buffer at 37°C for 24 h. The reaction was terminated by heating at 100°C for 10 min. Inactivated enzymes were removed by centrifugation at 4°C and 10,000 rpm for 10 min. Separation and purification of the solution fractions were carried out on a Sephadex G-75 column (2.6 × 100 cm, GE-healthcare) and eluted with 0.15 M NaCl at a rate of 0.4 ml/min. The eluent was collected every 2 ml and the distribution of total carbohydrate content was determined. The appropriate fractions were combined, and a Sephadex G-10 column was used for desalting.

### Analysis of Enzymatic Products

High-performance gel permeation chromatography (HPGPC) was used to assess weight-averaged molecular weights of the enzyme products with an LC-10Avp system (Shimadzu Company, Japan) and a TSK-gel G-3000 PWXL column (7.8 × 300 mm). The UPLC-MS method was performed using a Waters Acquity H-Class UPLC system linked to an ESI-MS mass spectrometer (amaZon speed ETD, Bruker, Germany) in the negative ion mode. Operating parameters were as follows: capillary voltage, 4.5 KV; capillary temperature, 200°C; nebulizer gas, 2 bar; dry gas, 6 l/min; scan range, 100–1000. RG oligosaccharide separation was carried out on an Acquity UPLC BEH Amide column (1.7 μm, 2.1 mm × 150 mm) at 35°C with a flow rate of 0.3 ml/min mobile phase. The mobile phase consisted of ACN and H_2_O in a ratio of 20/80 (v/v) for mobile phase A, 80/20 (v/v) for mobile phase B, and pH 3.0 200 mM ammonium formate/50 mM formic acid buffer for mobile phase C. The run time for oligosaccharide separation was 60 min, and the elution procedure was as follows: concentration of C remained at 5% during the entire elution process for 0–30 min, 0%-20% A; 30–31 min, 20–35% A; 31–40 min, containing 35% A; 40–41 min, 35–0% A; 41–50 min, containing 0% A.

## Results and Discussion

### Sequence Analysis of Two Rhamnogalacturonan Lyase Genes

**FIGURE 1:**
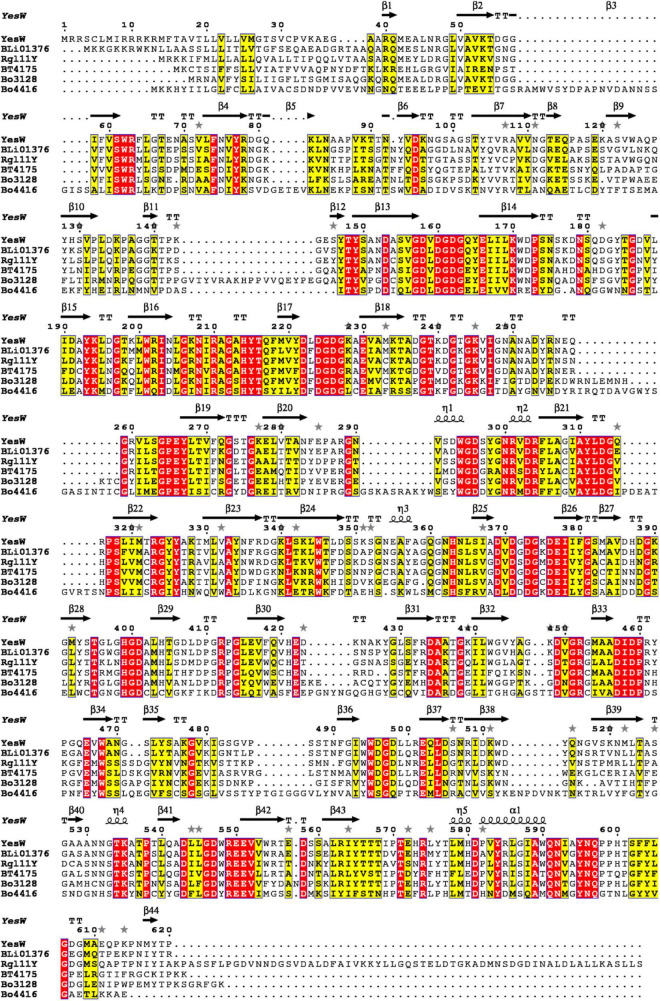
Multiple sequence alignments of Bo3128 and Bo4416 with known PL11 family endo-RGLs in the CAZy database from various microorganisms. GenBank accession numbers of the corresponding amino acid sequences are AAU40280.1 (BLi01376 from *Bacillus licheniformis* DSM 13), CAB12524.1 (YesW from *Bacillus subtilis* subsp. subtilis str. 168), ACL75117.1 (Rgl11Y from *Ruminiclostridium cellulolyticum* H10), and AAO79280.1 (BT4175 from *Bacteroides thetaiotaomicron* VPI-5482).

*Bacteroides ovatus* plays a vital role in the degradation of plant biomass and has been reported to contain a series of carbohydrate-active enzymes (Van Laere et al., 2000). According to the NCBI, *Bacteroides ovatus* ATCC 8483 contains two putative endo RGLs: Bo3128 (GenBank accession Number: ALJ47736.1) and Bo4416 (GenBank accession Number: ALJ49009.1), both belonging to the PL11 family. Bo3128 and Bo4416 may be involved in RG-I degradation, but their functions have yet to be characterized in detail. The sequence similarity between Bo3128 and Bo4416 is 25.1%, a relatively low value suggesting that Bo3128 and Bo4416 may play different roles in degrading RG-I polysaccharides. Multi-sequence alignments of Bo3128 and Bo4416 with other enzymes in the PL11 family (Figure 1) are, respectively: 52.9 and 31.8% to YesW, 52.3 and 33.6% to BLi01367, 49.1 and 31.2% to Rgl11Y and 48.9 and 31.6% to BT4175, and 42.8% and 31.3% to CtRGLf. These results indicate that both Bo3128 and Bo4416 are uncharacterized, new RGLs. The 3D-models of Bo3128 and Bo4416 have been predicted by using the SWISS-MODEL program with the RGL structure of YesW (PDB accession number: 2Z8R) from *Bacillus subtilis* strain 168 as the template and generated using Pymol (Supplementary Figure 1). In both structures, arginine (i.e., Bo3128-Arg467 and Bo4416-Arg516) and lysine (i.e., Bo3128-Lys549 and Bo4416-Lys615) are responsible for stabilizing carboxyl groups in these polysaccharides, and tyrosine (i.e., Bo3128-Tyr610 and Bo4416-Tyr675) stabilize the sugar ring by forming stacked interaction. Aspartic acid in Bo3128 (Asp414) and Bo4416 (Asp457), glutamic acid in Bo3128 (Glu435) and Bo4416 (Glu478), histidine in Bo3128 (His376 and His412) and Bo4416 (His419 and His454) coordinate with Ca^2+^ ions to likely enhance their enzymatic activities (Ochiai et al., 2007b).

### Expression and Purification of Recombinant Enzymes

**FIGURE 2:**
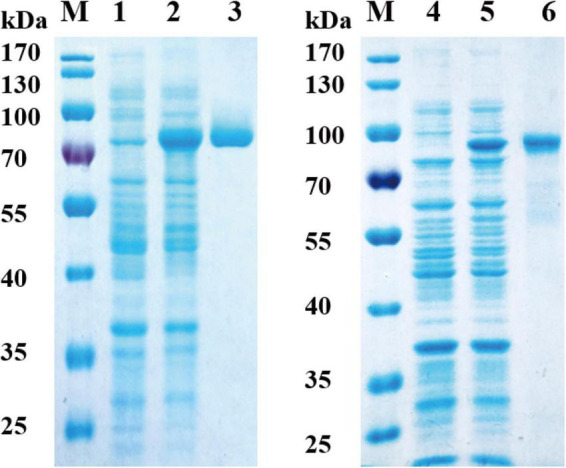
Analysis of recombinant Bo3128 and Bo4416 by SDS-PAGE. M: molecular weight standards. (1) Culture lysate of Bo3128 before IPTG induction. (2) Culture lysate of Bo3128 after IPTG induction. (3) Purified Bo3128. (4) Culture lysate of Bo4416 before IPTG induction. (5) Culture lysate of Bo4416 after IPTG induction. (6) Purified Bo4416.

The nucleotide sequences of RGL genes were amplified from the DNA of *Bacteroides ovatus* ATCC 8483. The two genes were cloned into the pET-28a (+) vector and overexpressed in *E. coli* BL21 (DE3). After purification by Ni-NTA column chromatography, 11.2 mg Bo3128 and 12.8 mg Bo4416 were obtained from 500 ml LB medium, and their specific activities against RG-I-AT were found to be 6.9 U/mg and 5.3 U/mg, respectively. The activities of Bo3128 and Bo4416 were determined with RG-I-AT. As the RG-I type pectin, RG-I-AT was obtained from the root of *Adenophora tetraphylla* (Thunb.) Fisch (Sun et al., 2019). The schematic diagram of RG-I-AT structure was shown in Supplementary Figure 2. SDS-PAGE indicated that the molecular weights of Bo3128 and Bo4416 are approximately 75 kDa and 80 kDa, respectively, values that are consistent with the predicted protein molecular weights (Figure 2). The total activities of Bo3128 and Bo4416 were 154.6 U/l and 153.7 U/l in a conventional bacterial cell culture. In order to increase production, a 5-liter bioreactor was used for high cell density fermentation. Protein concentrations obtained by fermentation were 187.2 mg/l and 205.4 mg/l, and the total activities for RG-I-AT were 1291.7 U/l and 1088.6 U/l. Following high density fermentation, total activities of these RGLs were significantly improved.

### Characterization of the Recombinant Enzymes

**FIGURE 3:**
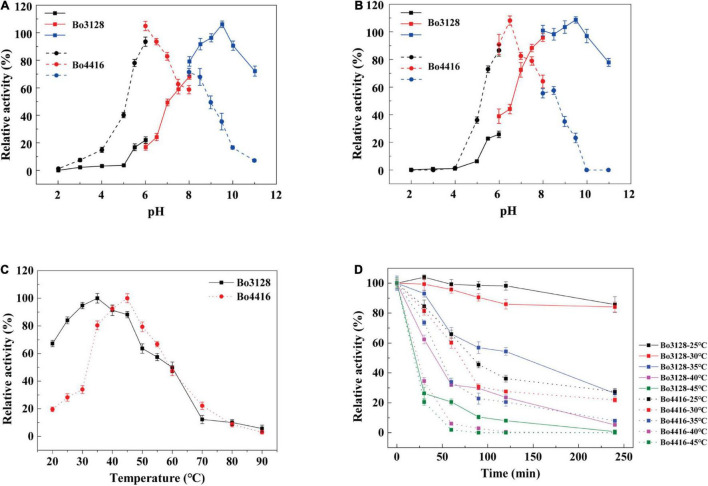
**(A)** Optimal pH of Bo3128 and Bo4416. **(B)** pH stability of Bo3128 and Bo4416. **(C)** Optimal temperature of Bo3128 and Bo4416. **(D)** Temperature stability of Bo3128 and Bo4416. Relative activity was calculated by setting maximum activity as 100%. Error bars represent the standard deviation of three independent determinations.

As shown in Figures 3A,B, Bo3128 exhibited the highest activity at pH 9.5, with good stability being maintained over the range of pH 8 to pH 11. These data showed that Bo3128 was stabile even under alkaline conditions similar to other enzymes in the PL11 family (Pagès et al., 2003; Dhillon et al., 2016). On the other hand, the optimal pH for Bo4416 was pH 6.5, and its stability could only be maintained within the pH range from pH 6.0 to pH 7.5. This indicated that Bo4416 can degrade substrates effectively under neutral and slightly acidic conditions. Interestingly, Bo3128 and Bo4416 from the same strain (*Bacteroides ovatus* ATCC 8483) exhibited acidophilic and basophilic properties, which may be related to the need for these species to adapt to more complex environments.

The optimum temperature range for Bo3128 activity was found to be between 25 and 45°C, whereas that for Bo4416 was between 30 and 50°C (Figure 3C). This is similar to some previously reported enzymes from *Bacteroides ovatus* ATCC 8483, e.g., BoXyl43A at 35°C (Zhou et al., 2020). The reason for their similar optimal temperature ranges may lie in the fact that *Bacteroides ovatus* is a member of the human intestinal bacteria, and the temperature of the human intestine is around 37°C. In terms of thermostability, Bo3128 retained 50% activity after 2 h at 35°C, making Bo3128 somewhat superior to Bo4416 that exhibited a half-life at 35°C of only 50 min (Figure 3D). Bo3128 could also maintain good temperature thermostability at lower temperatures (25°C and 30°C).

In the presence of 5 mM Ca^2+^, Mg^2+^ or Mn^2+^, the activity of Bo3128 increased by 1.5, 1.3 and 1.2 fold, respectively (Table 1). This indicates that upon addition of these divalent metal ions, activity of Bo3128 is increased. Ca^2+^ had a positive effect on Bo3128. However, 50 mM Ca^2+^ promoted the activity of Bo3128 as well as 5 mM Ca^2+^. This is conformable to PcRGL4A. The activity of PcRGL4A increased gradually at Ca^2+^ concentration of 1-10 mM and then reached a plateau at higher concentrations (Iwai et al., 2015). On the other hand, addition high concentrations of Hg^2+^ and Cu^2+^ completely inhibited activity of Bo3128. In contrast, Bo4416 has no enzyme activity in the absence of divalent metal ions (Ca^2+^, Hg^2+^, Mn^2+^), and Bo4416 still retains certain activity in the 50 mM divalent metal ions. The results indicates that Bo4416 requires divalent metal ions to function. Generally, RGLs in the PL11 family exert higher activities in the presence of Ca^2+^, and may even require calcium for activity. For instance, the activity of CtRGLf was enhanced by 1.5 fold in the presence of 3 mM Ca^2+^ (Dhillon et al., 2016), and Rgl11A (McKie et al., 2001) from *Pseudomonas cellulosa* had an absolute requirement for Ca^2+^.

**TABLE 1:** Effects of metal ions on Bo3128 and Bo4416 activities.

Metal ions or reagents	Relative activity (%)			
	Bo3128^a^		Bo4416^b^	
	5 mM	50 mM	5 mM	50 mM
None	100.0 ± 0.0	100.0 ± 0.0	–	–
NaCl	107.7 ± 1.6	56.4 ± 2.7	–	–
KCl	81.5 ± 3.2	35.3 ± 1.9	–	–
MgCl_2_	128.2 ± 4.3	97.1 ± 4.6	–	–
CaCl_2_	150.6 ± 2.9	139.7 ± 3.1	100.0 ± 0.6	90.4 ± 5.5
BaCl_2_	60.4 ± 3.8	25.1 ± 3.4	–	–
CuCl_2_	14.9 ± 0.9	–*^c^*	–	–
HgCl_2_	3.2 ± 0.4	–	143.0 ± 4.6	43.1 ± 3.6
MnCl_2_	117.2 ± 6.5	101.4 ± 6.2	103.0 ± 2.4	83.4 ± 4.2

*The experimental conditions were described in the text. Experiments were performed in triplicate, and data was expressed as mean the standard deviation. Statistical differences analysis was not performed.*

*^a^The relative activity of Bo3128 was calculated in the absence of metal as 100%.*

*^b^The relative activity of Bo4416 was in the presence of 5 mM CaCl_2_ as 100%.*

*^c^Not detected.*

From the Lineweaver-Burk plots, the Michaelis–Menten constant *K*_*m*_ values for Bo3128 and Bo4416 against RG-I-AT is were obtained: 0.56 ± 0.06 mg/ml and 0.27 ± 0.03 mg/ml., and The *k*_*cat*_/*K*_*m*_ values for Bo3128 and Bo4416 are 22.5 ± 1.6 ml⋅mg^–1^⋅s^–1^ and 35.1 ± 0.5 ml⋅mg^–1^⋅s^–1^, respectively (Supplementary Figure 3). These results show that the catalytic efficiency of Bo4416 is higher than that of Bo3128.

### Substrate Specificity of Bo3128 and Bo4416

We investigated the substrate specificity of Bo3128 and Bo4416 using pectin domains as RG-I-AT, RG-I-P, galactan, arabinan, HG, De-HG and RG-II (Supplementary Figure 2). As shown in Table 2, Bo3128 and Bo4416 displayed the greatest activity against RG-I pectin (RG-I-AT), indicating that the two enzymes could degrade RG-I pectin better than all other pectin domains tested, especially HG pectin that was very minimally degraded. HG pectin is usually modified with methyl groups (∼48% methyl-esterified HG) or side chain modifications as in RG-II, enzyme activity decreases significantly. In this regard, it appears that both Bo3128 and Bo4416 specifically act on RG-I pectin.

**TABLE 2:** Substrate specificity of Bo3128 and Bo4416.

Substrate	Monosaccharide composition (mol%)					Relative activity (%)*^a^*		Structural features
	GalA	Rha	Gal	Ara	Other sugars	Bo3128	Bo4416	
De-HG *^c^*	92.2	3.3	2.3	–	2.2	16.2 ± 2.2	15.6 ± 2.1	HG
HG (48% Methyl-esterified)^d^	76.7	3.3	14.7	1.7	3.6	– *^b^*	7.4 ± 1.9	HG
RG-II *^e^*	48.1	14.8	15.1	10.8	11.2	–	–	RG-II
RG-I-AT *^f^*	31.1	25.3	21.0	8.9	13.7	100.0 ± 2.0	100.0 ± 1.4	RG-I
RG-I-P *^g^*	47.2	21.9	25.8	1.3	3.8	35.7 ± 2.8	50.3 ± 1.3	RG-I + HG
Galactan *^h^*	25.9	19.0	51.3	2.8	1.0	30.8 ± 1.6	1.3 ± 0.2	Galactan + RG-I backbone
Arabinan *^i^*	8.3	1.4	13.3	74.1	2.9	14.5 ± 0.7	1.9 ± 0.3	Arabinan + RG-I backbone

*^a^Activity toward RG-I-AT was taken as 100%. The experiments were conducted in triplicate, and data was expressed as mean the standard deviation. Statistical differences analysis was not performed.*

*^b^Not detected.*

*^c^De-HG domain form the backbone via α-1,4-GalA linkages with no methyl-esterification (Yu et al., 2021).*

*^d^HG (48% Methyl-esterified, from Citrus Peel, Sigma) domain is formed by α-1,4-D-GalA polymerization and the degree of methylation is 48%.*

*^e^RG-II domain (Wu et al., 2018) has a linear backbone composed of (1→4)-linked-α-D-GalpA units, partly branched with oligosaccharide side chains.*

*^f^RG-I-AT is the RG-I type pectin extracted from the root of Adenophora tetraphylla (Thunb.) Fisch according to the method of Sun et al. (Sun et al., 2019).*

*^g^RG-I-P (from potato, Megazyme) is composed of RG-I and HG type pectin.*

*^h^Galactan (from potato, Megazyme) is constituted of (1→4)-linked β-D-Galp units, and contains the low content of RG-I backbone.*

*^i^Arabinan (from sugar beet, Megazyme) is composed of α-(1→5)-linked Araf residues, which can be further branched by α-L-Araf units at O-2 and/or O-3. Arabinan contains the low content of RG-I backbone.*

When compared to RG-I-AT, RG-I-P pectin is a poor substrate for both Bo3128 and Bo4416. This may be explained by considering that RG-I-P pectin is not a pure RG-I pectin, because it has a monosaccharide composition of 62% GalA and 20% Rha as well as containing a small amount of HG pectin. On the other hand, the content of GalA and Rha in RG-I-AT is about 1:1, indicating that this pectin is a relatively pure RG-I substrate. In addition, RG-I-AT contains both RG-I pectin main chain elements and side chains, while galactan and arabinan are RG-I pectin side chain domains. Both Bo3128 and Bo4416 showed the highest activity against RG-I-AT, and relatively low activity against galactan and arabinan. In this regard, Bo3128 and Bo4416 primarily degrade the main chain of RG-I pectin. Bo3128 exhibited weak activity against galactan and arabinan, which probably due to the low content of GalA and Rha residues present in galactan and arabinan. Compared with Bo3128, Bo4416 showed almost no activity against galactan and arabinan, suggesting that both arabinan and galactan side chains inhibited its activity.

### Insight Into the Mechanism of Action of Bo3128 and Bo4416

**FIGURE 4:**
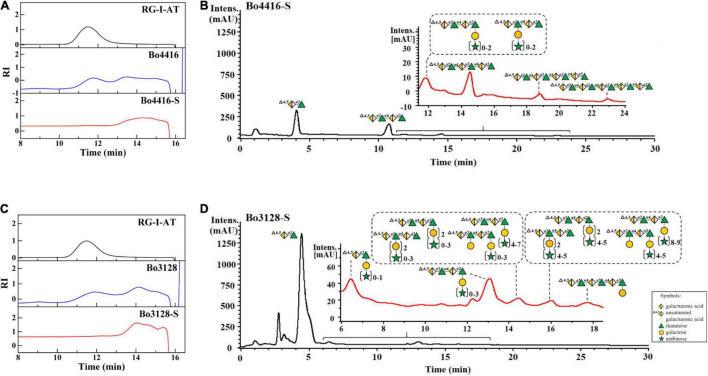
HPGPC and UPLC-MS analysis of enzymatic products of Bo3128 **(A,B)** and Bo4416 **(C,D)** released from RG-I-AT. RG-I-AT: RG-I-AT before enzymolysis; Bo3128 and Bo4416: enzymatic products of Bo3128 and Bo4416; and Bo3128-S and Bo4416-S: oligosaccharide fraction of Bo3128 and Bo4416.

Products from Bo3128- and Bo4416-mediated polysaccharide degradation were analyzed by using HPGPC with a TSK-GEL G3000PWXL column. As shown in Figure 4, RG-I-AT has a weight-average molecular weight of ∼32 kDa and shows a single, symmetric peak prior to enzymolysis. Following 24 h of enzyme action, a number of oligosaccharides were detected as reaction products (Figures 4A,C). The two RGLs may play function *via* an endo mode action. To determine the final major oligosaccharides products, the reaction products were further separated by using a Sephadex G-75 column that yielded oligosaccharide fractions Bo4416-S and Bo3128-S. The yield from Bo4416 degradation of RG-I to produce oligosaccharides was 32.4%, whereas the yield from Bo3128 was 62.4%.

UPLC-MS was used to further analyze the composition of oligosaccharide fractions. A series of unsaturated RG oligosaccharides (absent side chains) were detected in the Bo4416-S fraction, with the primary products being unsaturated RG disaccharides, tetrasaccharides, hexasaccharides, and octasaccharides (Figure 4B), along with a small amount of unsaturated oligosaccharides having single side chains (Supplementary Figure 4A). Analysis of Bo4416-S show that Bo4416 is an endo-RGL that mainly degrades regions in RG-I that contain no side chains. In addition to the presence of unsaturated RG disaccharides, the Bo3128-S fraction also contained unsaturated RG disaccharides and unsaturated RG tetrasaccharide substituted with galacto-oligosaccharides (Figure 4D) or galacto-oligosaccharides with arabino-oligosaccharide side chains. The unsaturated RG tetrasaccharide (^Δ4,5^GalA_1_-Rha_1_-GalA_2_-Rha_2_) could have galacto-oligosaccharides appended to the Rha_1_ or Rha_2_ residues (Supplementary Figures 4B–D). Our results indicate that Bo3128 is also an endo-RGL that can degrade RG-I pectin at regions that have no or short side chain substitutions.

In previous reports on RGLs, only a few studies had analyzed their primary products, but none had analyzed the composition of secondary products. For example, the primary product of YesW- and YesX- degraded RG-I from potato was an unsaturated RG disaccharide (Ochiai et al., 2007a). With AM-RG-I (RG-I without side chain) as substrate, BT4170 generated two main products: unsaturated RG disaccharide and unsaturated RG tetrasaccharide. On the other hand, BT4175 released an unsaturated RG hexaoligosaccharide, and BT4183 produced four unsaturated RG oligosaccharides with degrees of polymerization greater than or equal to tetrasaccharides (Luís, 2017). Against an apple rhamnogalacturonan, Rgl11A (McKie et al., 2001) cleaved an oligosaccharide comprising a backbone of RG hexasaccharides (Rha_1_-GalA_1_-Rha_2_-GalA_2_-Rha_3_-GalA_3_) in which Rha_1_ and Rha_3_ had galactose moieties attached, whereas the structure of other oligosaccharide products were not mentioned.

Furthermore, the primary products of BLi01367 (Silva et al., 2011), AsRglA (Yoshino-Yasuda et al., 2012), and Rgl11Y (Pagès et al., 2003) were never determined. To date, the structures and compositions of RGL oligosaccharide products have usually not been reported. Our research found that the smallest product of Bo3128- and Bo4416-mediated degradation of RG-I-AT is unsaturated RG disaccharide. The remaining oligosaccharide products of Bo4416 comprised a series of unsaturated RG oligosaccharides lacking side chains, whereas Bo3128 yielded a series of unsaturated oligosaccharide linked to galacto-oligosaccharides and arabino-oligosaccharides as side chains. In this regard, Bo4416 primarily degrades RG-I regions without side chain substitutions, whereas Bo3128 can degrade regions with some short side chain substitutions. Thus, the two endo-RGLs derived from *Bacteroides ovatus* ATCC 8483 are responsible for degrading somewhat different regions in RG-I. The degradation products of RG-I pectin by these enzymes are abundant, making both Bo4416 and Bo3128 useful in the preparation of rhamnogalacturonic oligosaccharides with varying degrees of polymerization and side chain content.

## Conclusion

Nowadays, enzymatic processes are crucial to green biotechnology. In this context, pectin-degrading enzymes are essential for production of rhamnogalacturonan oligosaccharides (Babbar et al., 2016). In our present study, we cloned, expressed and characterized two novel RGLs (Bo3128 and Bo4416) from *Bacteroides ovatus* ATCC 8483. Both enzymes displayed an endo-type mode of action against RG-I. Bo4416 primarily degraded RG-I-AT to form unsaturated RG oligosaccharides with different degrees of polymerization. In addition to producing unsaturated RG-disaccharides, Bo3128 also degraded RG-I-AT and produced a series of unsaturated RG-oligosaccharides with short side chains. With RG-I as substrate, product yields from Bo4416- and Bo3128-mediated degradation to RG oligosaccharides was 32.4 and 62.4%, respectively. Overall, the discovery of these two RGLs has significant potential in the enzymatic production of novel rhamnogalacturonan-based oligosaccharides.

## Data Availability Statement

The original contributions presented in the study are included in the article/Supplementary Material, further inquiries can be directed to the corresponding authors.

## Author Contributions

YZ and YY conceived and designed the research. WW, YW, HY, YL, GZ, and LZ performed the experiments. WW analyzed the data. YZ, YY, KM, and WW wrote and edited the manuscript. All authors reviewed the manuscript and read and approved the final manuscript.

## Conflict of Interest

The authors declare that the research was conducted in the absence of any commercial or financial relationships that could be construed as a potential conflict of interest.

## Publisher’s Note

All claims expressed in this article are solely those of the authors and do not necessarily represent those of their affiliated organizations, or those of the publisher, the editors and the reviewers. Any product that may be evaluated in this article, or claim that may be made by its manufacturer, is not guaranteed or endorsed by the publisher.

## Footnotes

1
www.cazy.org

2
http://www.cbs.dtu.dk/services/SignalP
