# Supplementary material for: Biochemical Characterization of Two Rhamnogalacturonan Lyases From Bacteroides ovatus ATCC 8483 With Preference for RG-I Substrates
Source: Front Microbiol. 2022 Jan 11;12:799875. doi: 10.3389/fmicb.2021.799875 (PMC8787155; doi:10.3389/fmicb.2021.799875)
Supplement: Supplementary file 1 [file Data_Sheet_1.docx]

***Supplementary Material***

**Supplementary Figure 1**

**
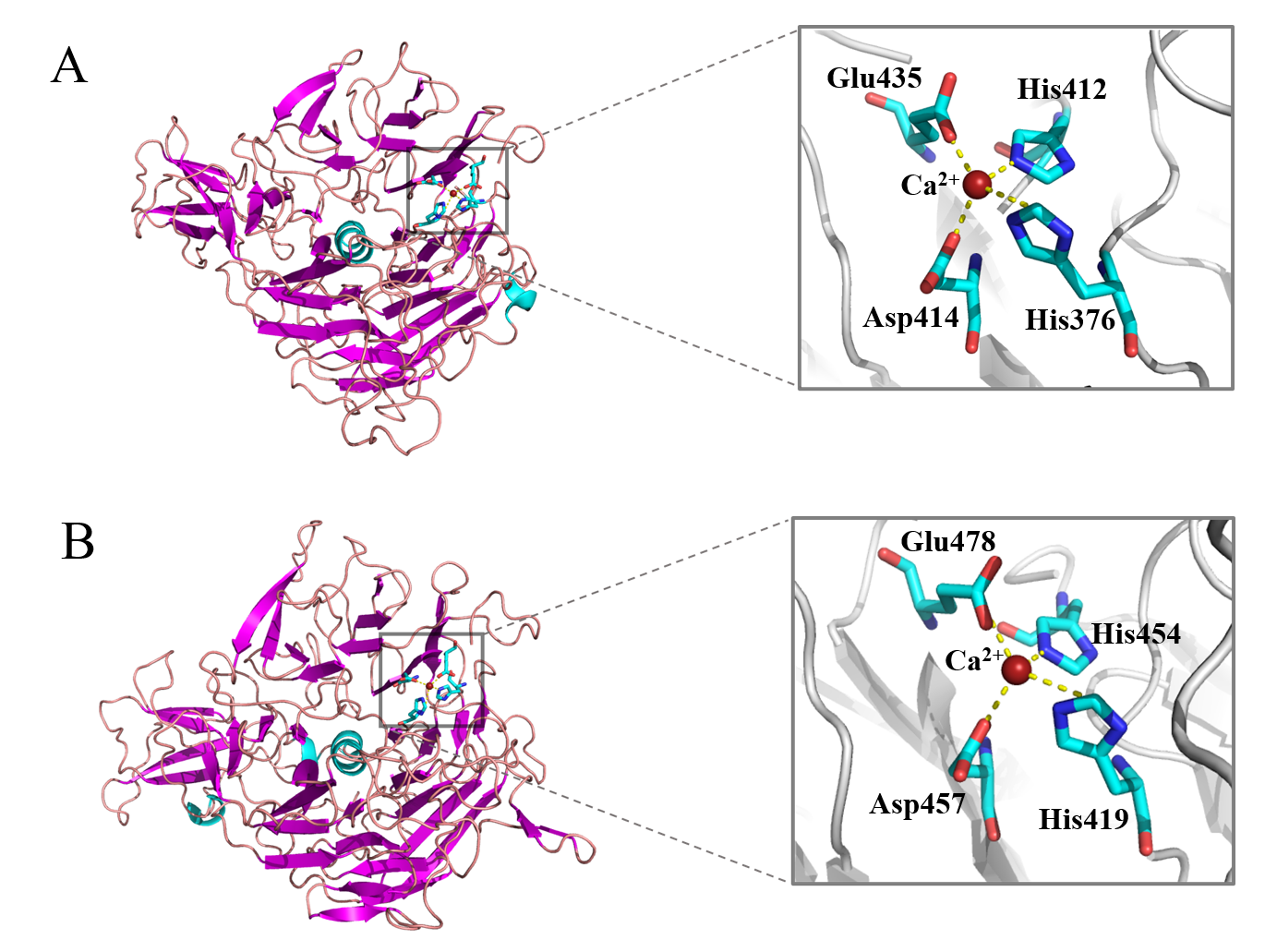
**

**Supplementary Figure 1.** 3D structural models of Bo3128 (A) and Bo4416 (B) were generated using Pymol. Parallel β-strands, linkers and α-helices are shown in magenta, orange and cyan, respectively. Asp (414-Bo3128 and 457-Bo4416), Glu (435-Bo3128 and 478-Bo4416), His (376-Bo3128 and 419-Bo4416) and His (412-Bo3128 and 454-Bo4416) residues are shown in cyan lines. The above referenced amino acid residues are all shown as sticks, with Ca^2+^ being displayed as a firebrick sphere.

**Supplementary Figure 2**


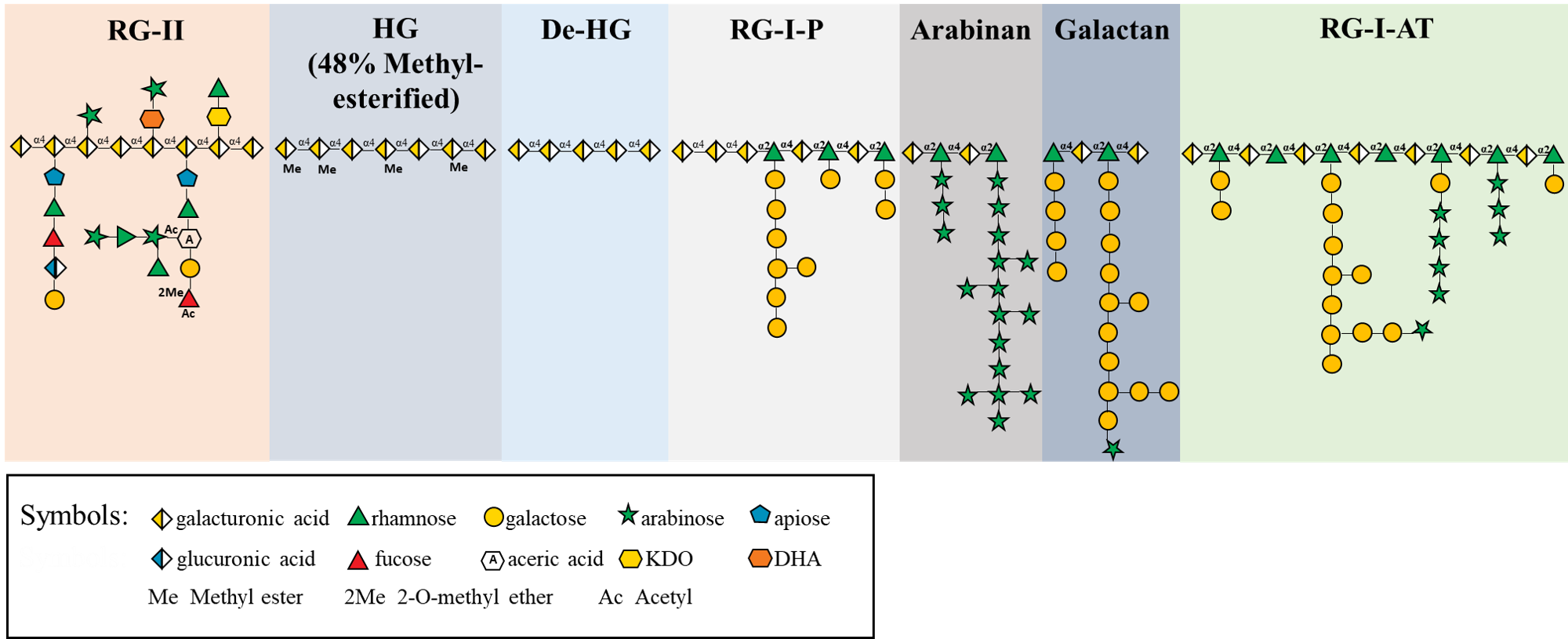


**Supplementary Figure 2.** Schematic of substrate structure showing the different polysaccharides highlighted with different colored backgrounds. The monosaccharide composition is represented according to the Symbol Nomenclature for Glycans.

**Supplementary Figure 3**

**
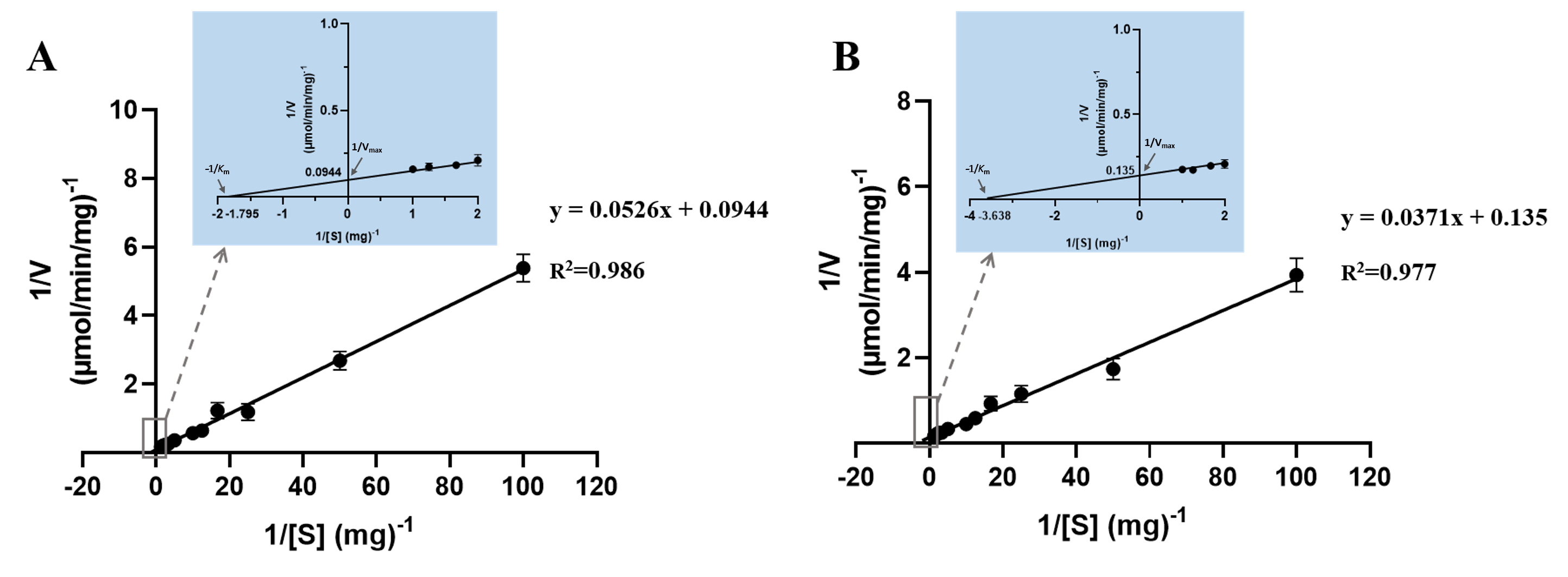
**

**Supplementary Figure 3. The Lineweaver-Burk plots of Bo3128 (A) and Bo4416 (B).**

**Supplementary Figure 4**


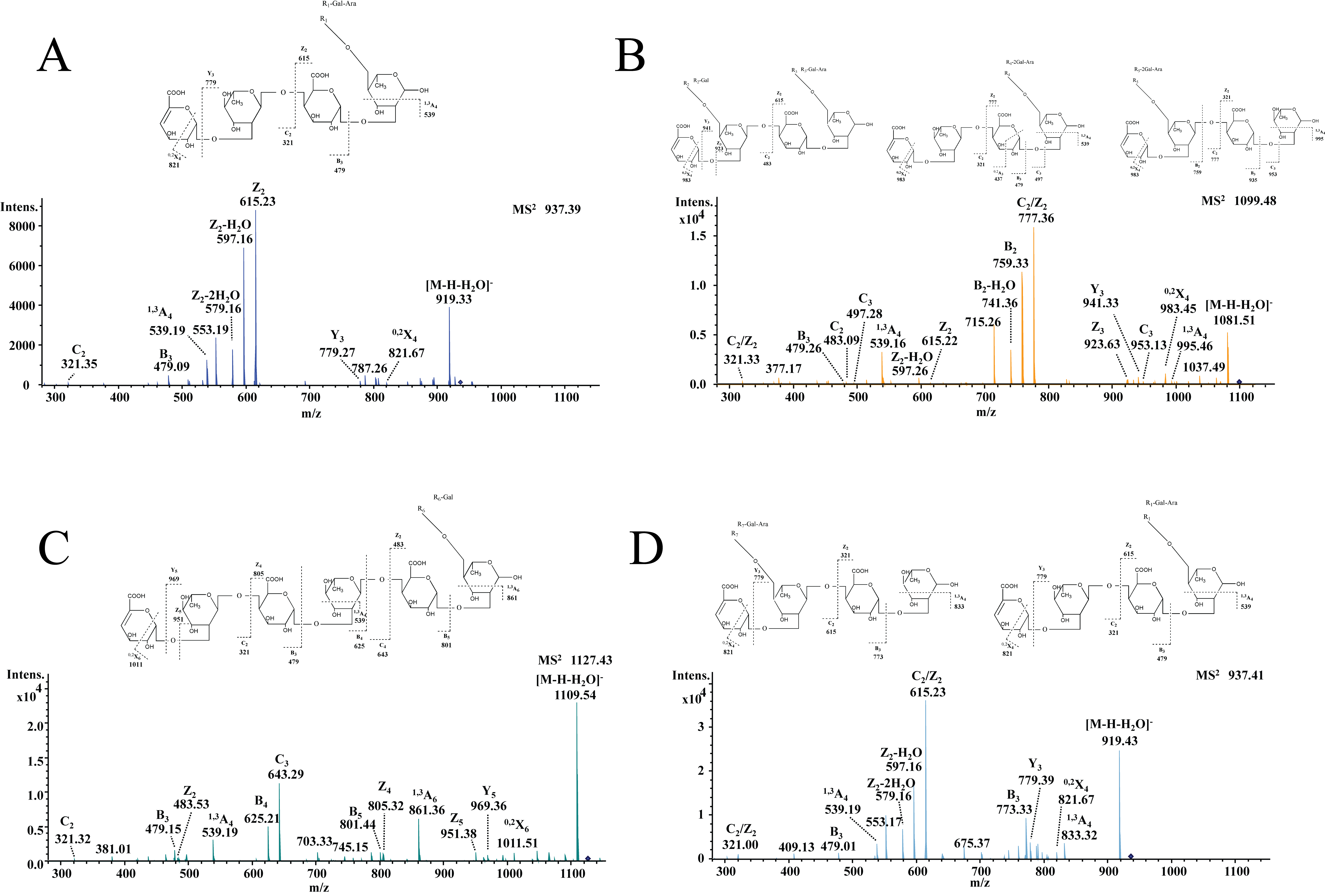


**Supplementary Figure 4.** MS^2^ spectrum of oligosaccharide products of Bo3128 and Bo4416. (A) Unsaturated RG tetrasaccharide with galactose and arabinose substitutions in Bo3128-S. (B) Unsaturated RG tetrasaccharide with two galactose and arabinose substitutions in Bo3128-S and Bo4416-S. (C) Unsaturated RG hexa-oligosaccharide with galactose and arabinose substitutions in Bo3128-S. (D) Unsaturated RG tetrasaccharide with galactose and arabinose substitutions in Bo4416-S.
